# Life expectancy improvement for multiple cure distributions

**DOI:** 10.1007/s13385-020-00229-y

**Published:** 2020-04-27

**Authors:** Shanoja Naik, Peter Adamic

**Affiliations:** Laurentian University, Sudbury, Canada

**Keywords:** Life expectancy, Multiple decrement, Competing risks, Hazard function, Cure distribution, Mixture model

## Abstract

In many circumstances, the increase in life expectancy when certain causes of death are eliminated is sought. These calculations are typically based on the assumption that the causes in question are simply omitted, which is equivalent to the causes being taken out of consideration, from the outset, with certainty. In this paper, we propose models whereby probability distributions for the cures of specific causes of death over time can be incorporated so as to more accurately predict the increase in life expectancy that would ensue. The theoretical results are applied to a real data set involving Diabetes and HIV-related deaths from Denver, Colorado, United States of America, between the years 1990 and 2015 inclusive.

## Introduction

Modeling the increase in life expectancy when a certain decrement^1^ is eliminated is an important statistic in many respects and, as noted by Beltran-Sanchez et al [3], is also an active area of demographic research. Actuaries, statisticians, demographers, engineers, sociologists, and econometricians all employ this concept when assessing the degree of influence certain causes of death (or failure, in a broader context) have on different populations (be they biological or not). The topic has been addressed in the literature from a wide array of different perspectives. Manuel et al [16] consider the issue of cause-deleted life expectancy that is modified to reflect what they call *health-related quality of life* factors. Somerville & Francombe [19] study the elimination of heart disease and cancer on mortality, as well as some of the accompanying consequences for life insurance. Mackenbach et al [14] investigate the gains in life expectancy after a cause of death is removed in light of the effect of related competing causes of death, and tackle several issues involved in the modeling and interpretation of these gains in life expectancy. Lai et al. [8] specifically consider the expected lifetime gains from the elimination of the human immunodeficiency virus (HIV), or HIV/AIDS. In Sect. 5 of this present article, a case study will be provided that applies the subsequent theory to a data set consisting of Diabetes and HIV-related deaths.

The concept of a *cause-deleted life expectancy improvement* (CDLEI), as found in Brown [5] and Adamic [1], is a statistic designed to quantify the increase in life expectancy if a certain cause of death is removed. The traditional approach to calculating the CDLEI is to remove the deaths caused by the failure in question from the life table (using an assumption or two, such as uniform distribution of decrements (UDD) and mutual independence between risks, as these random variables are not directly observable) and then to recalculate the life expectancies. One of the main drawbacks of this approach is that it is an either-or proposition: either the cause of death is allowed to remain perennially present, or it is taken out of consideration completely from the outset. Neither of these two extremes will necessarily correspond well with reality. To remedy this impasse, we will propose models that can incorporate probability distributions for the *cures* of the causes in question over time.

Accounting for possible cures in cause-deleted life expectancy calculations has several real-world applications. For example, if all of the life expectancies are known for each age, it is a fairly straightforward task to construct the entire life table from that information alone. Thus, if there are reasons to surmise that life expectancies will increase due to the emerging possibility of one or more cures for one or more causes of death, a new life table can be immediately created to reflect this reality. This is an especially important consideration for statistical entities that construct *cohort life tables*. Cohort life tables are typically built by the extrapolation of long term trends. Sometimes these trends are analyzed by cause of death. But the important thing to realize here is that these extrapolation methods will not adequately capture what we might call *cure shocks*, that is, significant breakthroughs in research that were not anticipated and that will result in immediate improvements in life expectancy.

Another example can be found in public health initiatives. Suppose a decision has to be made by public health officials regarding which cause of death should be granted research funding for a cure (and for how long). Although the cure of one risk, say cancer, would produce a life expectancy improvement well above a risk such as HIV/AIDS (since cancer accounts for so many more deaths), it may be the case that AIDS research has shown far more promising results in the interim than cancer research. This could very well imply that a cure for AIDS is substantially more likely than for cancer. If that were true, then a model that accounts for this would provide for a better basis of comparison between the two proposals in terms of expected lifetime gains.

It should be noted from the outset that our approach to modeling the cure of the cause itself is quite distinct from what is known as *cure modeling*, which is well established in the survival literature. For example, Tsodikov [20, 21] espouses a parametric cure model to estimate the proportion of long-term survivors. Dickman et al [6] consider regression models pertaining to relative survival. Li & Taylor [10], Lu & Ying [13], and Lu [11] consider semi-parametric cure models, with Peng & Dear [18] proposing a nonparametric mixture model. Overviews of various cure models utilized to date can be found in Friis & Chernick [7] and Othus et al. [17]. The subdiscipline of *cure fraction analysis* (cf. [2, 9]) also attempts to model survival based on the proportion cured as derived from an actual data set. Generally speaking, however, we may state that all of these methods involve fitting models, be they parametric or not, regression based or not, to data sets that include the actual times for which the subjects involved never experience the event of interest ([12]). But these methods are all different from modeling the probability of a cure of the cause of death *itself*, which, although much more difficult to do, is necessary in order to ultimately calculate the improved CDLEI, at least in the context we are considering.

Attempting to model an entire distribution for a future cure that has not yet occurred, is, admittedly, a task that is difficult and inherently imprecise. We may address this concern from a variety of different perspectives. First we note that, despite the difficulty inherent in the task, the revised CDLEI calculation allows for the possibility of a better estimator of the true CDLEI than when it is assumed a cure is certain and applied immediately. In other words, more information can be utilized. Second, experts in the field may be privy to knowledge regarding progress made for a specific risk, how long it took other risks at a similar stage of development to realize a full cure, or even the likelihood of a patent (or some other criteria) being approved to treat the particular risk (if the research has already progressed to that stage). The superiority of the new CDLEI estimator will very much depend on the nature and the quality of the information that is available regarding a cure. Third, it is entirely possible that a complete distribution for the time until cure need not be specified very precisely anyway: rather, a range of values for the cure distribution parameters may emerge within which, or outside of which, the CDLEI is not likely to change much. Or, as in the case where a comparison needs to be made (such as in the cancer/HIV example above), it may suffice to estimate the relative cure (analogous to the concept of *relative risk* in survival analysis) between the decrements instead of having to completely specify two distinct distributions.

The structure of the paper is as follows. In Sect. 2, we present some preliminary theory and nomenclature that will be used in subsequent sections. In Sect. 3, we summarize the cure model for the univariate case, and in Sect. 4 we develop the cure model in the multivariate setting, including a separate treatment for the bivariate and trivariate model before moving on the the general multivariate case. In Sect. 5 we consider a case study based on a real data set involving Diabetes and HIV-related deaths from Denver, Colorado, United States of America, between the years 1990 and 2015 inclusive, and we conclude with a summary and discuss possible future work in Sect. 6. The appendix gives a formal proof for Theorem 1, the most important theoretical result of the paper.

## Preliminaries and notation

Cause-deleted life calculations require the use of competing risks theory and notation. First, $h_x^{(j)}(t)$ would represent the hazard rate for risk *j* at time $x+t$, given a current age of *x*. We will sometimes drop the subscript *x* for notational convenience, but it will always be assumed that time starts at age *x*. Also note the implicit assumption that only one distinct cause can be responsible for any particular death. This assumption is germane to all of the theory presented in this paper. The negation of *j*, denoted $-j$, will refer to all risks other than *j*. Furthermore, ${_tp_x^{}}$ would represent the probability of surviving all risks up to time *t*. The following standard relationships are given without proof:

$$\begin{aligned} h_x^{(j)}(t) = \frac{f(t,j)}{_t p_x^{}} ,\,\,\,\,\,\, h_x^{}(t) = \sum _i h_x^{(i)}(t) = h_x^{(-j)}(t) + h_x^{(j)}(t) ,\,\,\,\,\, \, {_t {q_x^{}}} = \sum _j {_t q}_x^{(j)}, \end{aligned}$$

where $$f(t,j)dt \approx$$$$P \left\{ (t < T \le t+dt) \cap (J=j)\right\}$$ and where *J* designates the decrement. For example, $${_t q_x^{(j)}}$$ would represent the probability of failure for some subject aged *x* prior to time $$x+t$$ by cause *j* in the multiple risk forum.

The *complete life expectancy* (or simply the life expectancy) for an individual currently aged *x* quantifies the number of years the individual is expected to survive beyond *x*. This life expectancy will be denoted by $\mathring{e}_x$, with

$$\begin{aligned} \mathring{e}_x = E[T] = \int ^{\omega - x}_0 {_t p_x} \, dt, \end{aligned}$$

where this integral expression can be derived using a simple recursive formula (cf. [4]).

To calculate the life expectancy when cause *j* is deleted, we use the well-known expression,

$$\begin{aligned} \mathring{e}_x^{(-j)} = \int _0^{\omega - x} {_t p_x^{\prime \, (-j)}}dt , \end{aligned}$$

where ${_t p_x^{\prime \, (-j)}}$ is the “associated” survival function if cause *j* is eliminated as a competing risk, which can be expressed in terms of the cause-deleted hazard function:

$$\begin{aligned} _t p_x^{\prime \, (-j)} = \, {\text {exp}} \left( -\int _0^t h_x^{(-j)}(u)\, du \right) . \end{aligned}$$

Following the notation of Brown [5], when cause *j* is eliminated, the CDLEI for any age *x* equals $\mathring{e}_x^{(-j)} - \, \mathring{e}_x$. We also emphasize here that CDLEI calculations based directly on the hazard functions do not assume independence between the competing risks. This is a considerable advantage, as, in practice, many competing risks of death will be mutually dependent.

Some further notation from matrix theory is required in order to prove the results developed in subsequent sections. Let ${\mathbb {L}}$ be a field and let $A = [a_{ij}] \in {\mathbb {L}}^{m\times n}$ and $B = [b_{ij}] \in {\mathbb {L}}^{p\times q}$. Then the Kronecker product between *A* and *B* is denoted as $A\otimes B$, and is defined as

$$\begin{aligned} A\otimes B = [a_{ij} B] = \begin{bmatrix} a_{11}B&{}a_{12}B&{}\cdots &{}a_{1n}B\\ \vdots &{}\vdots &{}\vdots &{}\vdots \\ a_{m1}B&{}a_{12}B&{}\cdots &{}a_{mn}B \end{bmatrix} \in {\mathbb {L}}^{(mp)\times (nq)} \,, \end{aligned}$$

where the resultant matrix $A\otimes B$ is of dimension $(mp)\times (nq)$. We also propose the following Lemma, which will be used in a subsequent proof.

### Lemma 1

*Let*$A = [a_{ij}] \in {\mathbb {L}}^{m\times m}$*and*$B = [b_{ij}] \in {\mathbb {L}}^{m\times m}$. *Then their Kronecker product can be converted to a matrix multiplication as follows:*

2.1 $$\begin{aligned} A\otimes B = [A\otimes I_m ]\,[I \otimes B_m] \,, \end{aligned}$$

*where*$I_m$*is an identity matrix of orderm*. *Also*$[A\otimes I_m ]$*and*$[I_m \otimes B]$*are commutative*. $\square$

## Cure model for the univariate case

In this section, we summarize the univariate model. Let $T_c$ be the future cure time of risk *j*. By cure we mean that risk *j* is no longer a potential candidate risk that can be responsible for failure. Note that the time until cure can itself be described using a survival function, since we would be modeling the time until some event of interest, in this case cure, occurs. Without loss of generality, we suppress the age subscript *x* here for notational convenience. Let $F_c(t) = P(T_c \le t)$ where $F_c(t)$ would quantify the probability that a cure has been found for cause *j* by time *t*. With the probability distribution for the cure of cause *j* specified, we can define the hazard function for this process. The new hazard function, $h^*(t)$ say, is the hazard rate accounting for all causes of death when cure for cause *j* can be found with probability $F_c(t)$ by time *t*. The new hazard will be a mixture of the hazard function without *j* with the hazard of all risks in toto, as follows:

3.1 $$\begin{aligned} h^*(t) = F_c(t) h^{(-j)}(t) + [1-F_c(t)] h^{}(t) \, , \end{aligned}$$

since $F_c(t)$ is the probability that cause *j* is in a cure state at the future time *t*. Equation (3.1) is an exact relationship, although the actual $F_c(t)$ that is chosen will depend on what assumptions are employed regarding the probability of cure.

It is instructive to rearrange Eq. (3.1) as follows. Using the property that $h^{ (-j)}(t) +h^{ (j)}(t) = h^{ }(t)$, the new hazard function can be expressed as

3.2 $$\begin{aligned} h^*(t) = h^{}(t) - F_c(t) h^{ (j)}(t). \end{aligned}$$

This is an intuitive result. The new hazard function, $h^*(t)$, is the total hazard $h^{}(t)$, minus the expected hazard for cause *j* reflecting the probability that cause *j* is no longer present at future time *t*.

The revised life expectancy can now be determined. For any age *x* and cumulative probability distribution of cure $F_c(t)$, the following sequence of arguments engender a single equation for the revised CDLEI for any age *x*:

3.3 $$\begin{aligned} \text {CDLEI}(x)= \,& {} \mathring{e}_x^{ *} - \mathring{e}_x \nonumber \\= & {} \int _0^{\omega - x} e^{-\int _x^{x+t} h^{}(u) - F_c(u) h^{(j)}(u) du} dt \, \,- \, \int _0^{\omega - x} e^{-\int _x^{x+t} h^{}(u) du} dt \nonumber \\= & {} \int _0^{\omega - x} {_t p_x^{}} \cdot \, \left( {e^{\int _x^{x+t} F_c(u) h^{(j)}(u)du}} - 1\right) dt . \end{aligned}$$

It is important to note that our model can handle both increasing or decreasing rates, over time, in life expectancy. However, removal of causes of death, in and of themselves, can only increase life expectancy. For example, even if heart disease improvements are realized over time, it is still the case that removal of heart disease as a cause of death can only increase life expectancy. In this case, the gains in life expectancy will be less if there are already improvements in heart disease mortality rates.

## The multivariate cure model

### The bivariate extension of the cure model

For the bivariate extension of the model, let $F_{j_1,c}(t)$ and $F_{j_2,c}(t)$ represent the probability that a cure has been found, by time *t*, for causes $j_1$ and $j_2$ respectively. We make the assumption that time of cure for the two causes are independent random variables, but not that the decrements associated with these cures are independent. With these probability functions for the cures of causes $j_1$ and $j_2$, we can define a new hazard function as a mixture of: (1) the hazard function without both $j_1$ and $j_2$, that is, $h^{(-j_1-j_2)}(t)$; (2) the hazard function without cause $j_2$ only, or $h^{(-j_2)}(t)$; (3) the hazard without cause $j_1$, or $h^{(-j_1)}(t)$ only; and (4) the hazard of all risks in aggregate, which is $h^{}(t)$. Temporarily suppressing the subscript *x*, cure designator *c*, and time variation *t* for notational ease, by letting $F_{j_1,c}(t) = F_{j_1}$ and $F_{j_2,c}(t) = F_{j_2}$, the new hazard function for the bivariate case is as follows:

4.1 $$\begin{aligned} h^*_2 =F_{j_2} F_{j_1} h^{(-j_2-j_1)} + F_{j_2}(1-F_{j_1}) h^{(-j_2)} + F_{j_1}(1-F_{j_2}) h^{(-j_1)} + (1-F_{j_1})(1-F_{j_2}) h^{ }. \end{aligned}$$

As in the univariate case, this equation can be expressed in a more intuitive form. Now, since $h^{(-j_2-j_1)} = h^{} - h^{(j_2)} - h^{(j_1)}$, and $h^{(-j_2)} = h^{} - h^{(j_2)}$, and $h^{(-j_1)} = h^{} - h^{(j_1)}$, substitution into Equation (4.1) yields

4.2 $$\begin{aligned} h^*_2 = h^{} - F_{j_2} h^{(j_2)} - F_{j_1} h^{(j_1)} , \end{aligned}$$

which is an intuitive result analogous to Equation (3.2). Interestingly, we can also obtain $h^*_2$ by exploiting a direct relationship with the univariate hazard $h^*_1$ from the previous subsection as follows:

4.3 $$\begin{aligned} h^*_2= \,& {} \left[ h^{(-j_2-j_1)} F_{j_1}+ h^{(-j_2)} (1- F_{j_1}) \right] F_{j_2}+ \left[ h^{(-j_1)} F_{j_1} + h^{ } (1- F_{j_1}) \right] (1- F_{j_2}) \nonumber \\= \, & {} {\tilde{h}}_1 F_{j_2} + h^*_1 (1- F_{j_2}), \end{aligned}$$

where ${\tilde{h}}_1 = h^{(-j_2-j_1)} F_{j_1} + h^{(-j_2)} (1-F_{j_1})$. Thus, a convex combination of $F_{j_1,c}(t)$, as exhibited in $h^*_1$, and by utilizing the hazards $h^{(-j_2-j_1)}$ and $h^{(-j_2)}$, shows a recurring pattern of combinations of hazards and cure functions. From Equation (4.3), it is evident that $h^*_2$ also has a convex combination in terms of $F_{j_2}= F_{j_2,c}(t)$, the probability of cure for cause $j_2$ by time *t*. The pattern will be utilized in the ensuing theory.

It is instructive to express our results for the new hazard function for the univariate and bivariate cases already developed in matrix/vector form, as follows:

4.4 $$\begin{aligned} h^*_1= & {} (h^{ (-j_1)}, h^{ }) \begin{bmatrix} F_{j_1}&{}0\\ 0&{} 1-F_{j_1} \end{bmatrix} (1,1)'={\bar{h}}_1{{{{\mathbf{F}}_{\mathbf{1}}}}} \mathbf{1 }_{2\times 1}' \end{aligned}$$

4.5 $$\begin{aligned} h^*_2=\, & {} (h^{ (-j_2-j_1)},h^{ (-j_2)},h^{ (-j_1)}, h^{ }) \begin{bmatrix} F_{j_2}&{}0\\ 0&{} 1-F_{j_2} \end{bmatrix} \otimes \begin{bmatrix} F_{j_1}&{}0\\ 0&{} 1-F_{j_1} \end{bmatrix} (1,1, 1,1)' \nonumber \\= \, & {} {\bar{h}}_2 {{{\mathbf{F}_{\mathbf{2}}}}} \otimes {{{{\mathbf{F}}_{\mathbf{1}}}}} {{{\mathbf{1}'}_{{\mathbf{4}}\times {\mathbf{1}}}}}, \end{aligned}$$

where ${\bar{h}}_1$ and ${\bar{h}}_2$ are the appropriate hazard vectors for corresponding dimensions respectively. This matrix representation is favorable to the higher dimensional cure model. When we consider two cures (the bivariate model), there are $2^2 =4$ terms existing in the expression for the hazard. For higher dimensions, the number of terms in the hazard function $h_{i}^*, i=1,2, \ldots$, increases by powers of two. Motivated by the relationships demonstrated between the hazards and cure probabilities presented thus far, we now propose a general framework for an arbitrary number of cures, *n*, and an accompanying proof.

### The *n*-dimensional cure model

Let $F_{j_1}, F_{j_2},\cdots , F_{j_n}$ denote the probabilities that a cure has been found for causes $j_1,j_2,\cdots , j_n$ respectively by time *t*, and assume that the cure time random variables are all mutually independent for each and every cause of death. Let $h^{(j_1)}, h^{(j_2)}, \cdots , h^{(j_n)}$ denote the hazard functions with causes $j_1,j_2,\cdots , j_n$ respectively. For each distribution $F_{j_k},k=1,2,\cdots ,n$, we can create diagonal matrices of order 2 as follows:

4.6 $$\begin{aligned} {{\mathbf{F}}_{{\mathbf{k}}}}=\, \begin{bmatrix} F_{j_k}&{}0\\ 0&{} 1-F_{j_k} \end{bmatrix} , k=1,2,\cdots , n. \end{aligned}$$

For the *n*-dimensional cure model, there exists $2^n$ terms in the expansion of the hazard function, mimicking the expansion procedure previously done. By means of matrix notation, we define the hazard vector ${\bar{h}}_n$, which consists of hazards of individual cures and total cure. To formulate the vector of hazards, we should have $n \atopwithdelims (){n-1}$ of them assigned a combination of two of the $j_k's$. For example, $h^{(-j_{n}-j_{n-1})}, h^{(-j_{n-1}-j_{n-2})}, ..., h^{(-j_{3}-j_{2})}$, and for $n \atopwithdelims (){n-2}$ of them assign a combination of three of the $j_k's$ such as

$$\begin{aligned} h^{(-j_{2^n}-j_{2^n-1}-j_{2^n-2})}, h^{(-j_{n-1}-j_{n-2}-j_{n-3})},...,h^{(-j_4-j_{3}-j_{2})}, \end{aligned}$$

and finally one of the kind $h^{(-j_{n}-j_{n-1}-\cdots -j_{1})}$ and $h^{}$ in total. There should be $2^n$ terms of hazards to be found in the vector ${\bar{h}}_n$. For notational ease, we have chosen to use

$$\begin{aligned} i_{2^n}= & {} \,-j_{n}-j_{n-1}-\cdots -j_2-j_{1}\\ i_{2^n-1}= & {} \,-j_{n}-j_{n-1}-\cdots -j_{2}\\ \vdots \\ i_{3}= & {} \, -j_2\\ i_2= & {} \,-j_{1}\\ i_1= & {} \, \, \, \textit{all risks}, \end{aligned}$$

so that, the vector of hazards ${\bar{h}}_n$ has dimension $1 \times 2^n$ and is defined as

4.7 $$\begin{aligned} {\bar{h}}_n = (h^{(i_{2^n})},h^{(i_{2^{n}-1})} , \cdots , h^{(i_1)}). \end{aligned}$$

Also, we define ${\mathbf{1}}_{2^n \times 1}$ as a $2^n \times 1$ vector of ones. Using these precisions, we can obtain a general result for *n*-dimensional cure model.

#### Theorem 1

*Let*$F_{j_k}, k=1,2, ...,n$*be the cumulative cure probabilities forncompeting risks*$(j_k), k=1,2,\cdots ,n$*and*$F^n$*represents the Kronecker product ofndiagonal matrices of order* 2 *as in Equation* (4.6) *such that*

4.8 $$\begin{aligned} F^n = {{\mathbf{F}}_{{\mathbf{n}}}}\otimes {{\mathbf{F}}_{{\mathbf{n-1}}}} \otimes \cdots \otimes {{\mathbf{F}}_{\mathbf{2}}} \otimes {{\mathbf{F}}_{\mathbf{1}}}. \end{aligned}$$

*Utilizing the vector of hazards*${\bar{h}}_n$*of dimension*$1 \times 2^n$, *and be a column vector of ones*${\mathbf{1}}'$*with dimension*$2^n \times 1$, *the new hazard function is denoted by*$h_n^*$*and can be expressed in the form*

4.9 $$\begin{aligned} h_n^*={\bar{h}}_n F^n {\mathbf{1}}_{2^n\times 1}' \end{aligned}$$

*which has*$2^n$*terms of products of combinations of hazards*$h^{(.)}$*and independent cumulative cure probabilities*$F_{(.)}$.

Note that although the hazard function for the *n*-dimensional cure model can assume different forms, Equation (4.9) is perhaps the most basic way to express it. Now, the recurrence relation connecting successive models of higher dimension can be expressed in the form

4.10 $$\begin{aligned} h_{n}^* = {\tilde{h}}_{n-1} F_{i_{n}} + h_{n-1}^* [1- F_{i_{n}} ], \end{aligned}$$

where ${\tilde{h}}_{n-1}=(h^{(r_{2^{n-1}})},h^{(r_{2^{n-2}})},\cdots , h^{(r_{1})})$ and $h_{n-1}^* = {\tilde{h}}_{n-2}F^{n-2}\mathbf{1}_{2^{n-1} \times 1}$ with $F^{n-2}$ as the $n-2$ Kronecker products of 2 dimensional matrices of cure probabilities. A proof of the theorem, by means of principle of mathematical induction, is provided in the appendix.

Using the property of total hazards $h_x^{} = \sum _j h_x^{(j)}$, we obtain a more simplified form of the combined hazard as

4.11 $$\begin{aligned} h_m^*= h^{} - \sum _{k=1}^m F_{i_k} h^{(i_k)} \,. \end{aligned}$$

The multivariate extension of the revised life expectancy can now be determined in a simple form based on the survival probability and combined form of hazards and cures. For any age *x*, and cumulative probability distribution of cures $F_k, k=1,2,\cdots ,n$, with corresponding hazards $h^{(k)}, k=1,2,\cdots ,n$, with the total hazard $h^{}$, the revised CDLEI for any age can be written in the following form:

4.12 $$\begin{aligned} \text {CDLEI}(x)= & {} \int _0^{\omega -x} \mathrm{e}^{-\int _x^{x+t} [h^{}(u) \,-\, \sum _{k=1}^n F_k(u) h^{(k)}(u)] du} dt \,- \int _0^{\omega -x} \mathrm{e}^{-\int _x^{x+t} h^{}(u) du} dt \nonumber \\= & {} \int _0^{\omega -x} {_t p_x^{}}\left\{ \prod _{k=1}^n \mathrm{e}^{-\int _x^{x+t}F_k(u) h^{(k)}(u) du} -1\right\} dt. \end{aligned}$$

## Case study

In this section we illustrate the models proposed in this paper using a real data set. The data set gives the number of deaths from birth to age 109 for the citizens of Denver, Colorado, USA, between the years 1990 and 2015 inclusive for all causes of death, those that results from Diabetes, and those that result from contracting the human immunodeficiency virus (HIV). The data were obtained from the Colorado Department of Public Health and Environment. Although the data are available for each age, in the interest of space, we present it here in decennial intervals only. The data are summarized in Table 1. Some ages have such a small number of deaths that the results were not made available for reasons of anonymity. Two noteworthy attributes pertaining to the data include: (a) Diabetes-related deaths account for approximately 1.5% of the total number of deaths, or about 1 in every 67 deaths; (b) HIV-related deaths account for approximately 0.5% of the total number of deaths, or about 1 in every 200.

**Table 1:** Summary of the Denver HIV data

Age interval	Total Deaths	Diabetes Deaths	HIV Deaths
0-9	2,207	*ns*	*ns*
10-19	715	*ns*	*ns*
20-29	2,467	*ns*	*ns*
30-39	4,575	57	122
40-49	7,660	106	233
50-59	10,933	220	140
60-69	15,385	343	36
70-79	23,908	450	*ns*
80-89	30,646	439	*ns*
90-99	14,967	110	*ns*
100-109	879	*ns*	*ns*

*ns*: negligibly small number

To model the CDLEI (from age 0) for these data, we will employ a nonparametric approach. For this approach, we will estimate three functions: first the all-risks survival function, *S*(*t*), and then the associated survival functions of $S^{(-Diab)}(t)$ and $S^{(-HIV)}(t)$, where *Diab* is shorthand for *Diabetes*. Since we are modeling from time 0, note that $S^{(-j)}(t) = {_t p_x^{\prime \, (j)}}$. Specifically, the method that was used was a nonparametric generalized Kaplan-Meier estimator:

$$\begin{aligned} {_t p_x^{\prime \, (j)}} = \prod _{i: t_{ij}<t} \left( 1 - \frac{d_{ij}}{n_{ij}} \right) , \end{aligned}$$

where $t_{ij}$ represents the failure times for failures due to cause *j*, $n_{ij}$ the total number at risk just prior to each time point $t_{ij}$, and $d_{ij}$ the number of deaths at time point $t_{ij}$ by cause *j*.

Since this paper focuses on the implementation of cure distributions to more accurately predict the increase in CDLEI, it is advantageous here to consider different forms for the cure function itself. A dominant characteristic that would distinguish different forms for the cure function would be whether or not the cure of the cause of death would increase over time, remain constant, or decrease over time. The two-parameter Weibull distribution is good choice to consider here, due to it’s popularity in survival analysis, it’s inherent flexibility, and it possessing a hazard function that can monotonically increase, remain constant, or monotonically decrease over time, depending on the value of the shape parameter. We therefore make the following assumptions for the two cure distributions:

1. $${F}_{Diab}(t) = 1 - e^{-{(\lambda _1 t)}^{\alpha _1}}, \, \text {for} \, t \ge 0, \lambda _1, \alpha _1 > 0$$

2: $${F}_{HIV}(t) = 1 - e^{-{(\lambda _2 t)}^{\alpha _2}}, \, \text {for} \, t \ge 0, \lambda _2, \alpha _2 > 0.$$

The Weibull hazard function is $h(t) = f(t)/S(t) = \alpha \lambda ^{\alpha } t^{\alpha - 1}$. Now, $\frac{d}{dt} h(t) = \alpha (\alpha - 1) \lambda ^{\alpha } t^{\alpha - 2}$. From this result we can see that if $\alpha > 1$, then the hazard is monotonically increasing. In this case, the probability of obtaining a cure increases as time goes on *and* at an increasing rate. If, on the other hand, $\alpha = 1$, then the hazard is constant, implying that the probability of obtaining a cure does not improve with time (other than the fact that more time has elapsed, of course). This is hopefully not surprising, since when $\alpha = 1$, the Weibull distribution reduces to the exponential distribution, which has the famed *memoryless* property. Finally, if $\alpha < 1$, then the hazard is monotonically decreasing. In this case, the probability of realizing a cure for a cause of death would increase with time (as it must since *F*(*t*) is a non-decreasing function) but at a decreasing rate. We consider the first scenario to be the most realistic, since as time goes on, the impact of research and technology should at least make the probability of cure more likely than at an earlier point in time.

**Table 2:** CDLEI(0) Results for Select $\lambda _1$ and $\lambda _2$ Values

$\lambda _1$	$\lambda _2$	*Weibull*	*Weibull*	*Weibull*	*Weibull*
		$(\alpha _1 = \alpha _2 = 1)$	$(\alpha _1 = \alpha _2 = .5)$	$(\alpha _1 = \alpha _2 = 2)$	$(\alpha _1 = .5, \alpha _2 = 2)$
0.000	0.000	0.00000	0.00000	0.00000	0.00000
0.005	0.000	0.05548	0.08093	0.02396	0.08093
0.010	0.000	0.09337	0.10241	0.07717	0.10241
0.020	0.000	0.13706	0.12485	0.15590	0.12485
0.040	0.000	0.16758	0.14556	0.17681	0.14556
0.060	0.000	0.17476	0.15569	0.17725	0.15569
0.080	0.000	0.17656	0.16165	0.17727	0.16165
0.100	0.000	0.17705	0.16552	0.17727	0.16552
$\infty$	0.000	0.17727	0.17727	0.17727	0.17727
0.000	0.010	0.04073	0.04883	0.02981	0.03133
0.005	0.010	0.09630	0.12837	0.05380	0.11084
0.010	0.010	0.13425	0.14989	0.10708	0.13235
0.020	0.010	0.17800	0.17237	0.18591	0.15482
0.040	0.010	0.20857	0.19311	0.20685	0.17556
0.060	0.010	0.21575	0.20325	0.20728	0.18570
0.080	0.010	0.21756	0.20923	0.20730	0.19167
0.100	0.010	0.21805	0.21310	0.20730	0.19554
$\infty$	0.010	0.21827	0.22487	0.20730	0.20730
0.000	0.020	0.06178	0.05831	0.06778	0.06930
0.005	0.020	0.11739	0.13925	0.09180	0.14891
0.010	0.020	0.15536	0.16078	0.14515	0.17045
0.020	0.020	0.19915	0.18326	0.22408	0.19295
0.040	0.020	0.22974	0.20402	0.24505	0.21372
0.060	0.020	0.23693	0.21416	0.24548	0.22387
0.080	0.020	0.23873	0.22014	0.24550	0.22985
0.100	0.020	0.23922	0.22401	0.24550	0.23372
$\infty$	0.020	0.23944	0.23579	0.24550	0.24550
0.000	0.030	0.07283	0.06451	0.08196	0.08201
0.005	0.030	0.12846	0.14546	0.10599	0.16312
0.010	0.030	0.16645	0.16700	0.15936	0.18467
0.020	0.030	0.21025	0.18949	0.23831	0.20717
0.040	0.030	0.24085	0.21025	0.25928	0.22794
0.060	0.030	0.24804	0.22039	0.25972	0.23810
0.080	0.030	0.24984	0.22637	0.25974	0.24408
0.100	0.030	0.25034	0.23024	0.25974	0.24795
$\infty$	0.030	0.25056	0.24202	0.25974	0.25974
0.000	0.060	0.08369	0.07850	0.08601	0.08606
0.005	0.060	0.13933	0.15485	0.11004	0.16717
0.010	0.060	0.17733	0.17639	0.16340	0.18871
0.020	0.060	0.22115	0.19888	0.24236	0.21122
0.040	0.060	0.25176	0.21965	0.26333	0.23199
0.060	0.060	0.25895	0.22980	0.26377	0.24214
0.080	0.060	0.26075	0.23578	0.26379	0.24813
0.100	0.060	0.26124	0.23965	0.26379	0.25200
$\infty$	0.060	0.26146	0.25143	0.26379	0.26379
0.000	$\infty$	0.08603	0.08603	0.08603	0.08603
0.005	$\infty$	0.14168	0.16719	0.11006	0.16719
0.010	$\infty$	0.17968	0.18874	0.16343	0.18874
0.020	$\infty$	0.22349	0.21124	0.24238	0.21124
0.040	$\infty$	0.25410	0.23202	0.26336	0.23202
0.060	$\infty$	0.26129	0.24217	0.26379	0.24217
0.080	$\infty$	0.26310	0.24815	0.26381	0.24815
0.100	$\infty$	0.26359	0.25203	0.26381	0.25203
$\infty$	$\infty$	0.26381	0.26381	0.26381	0.26381

**Fig. 1:**
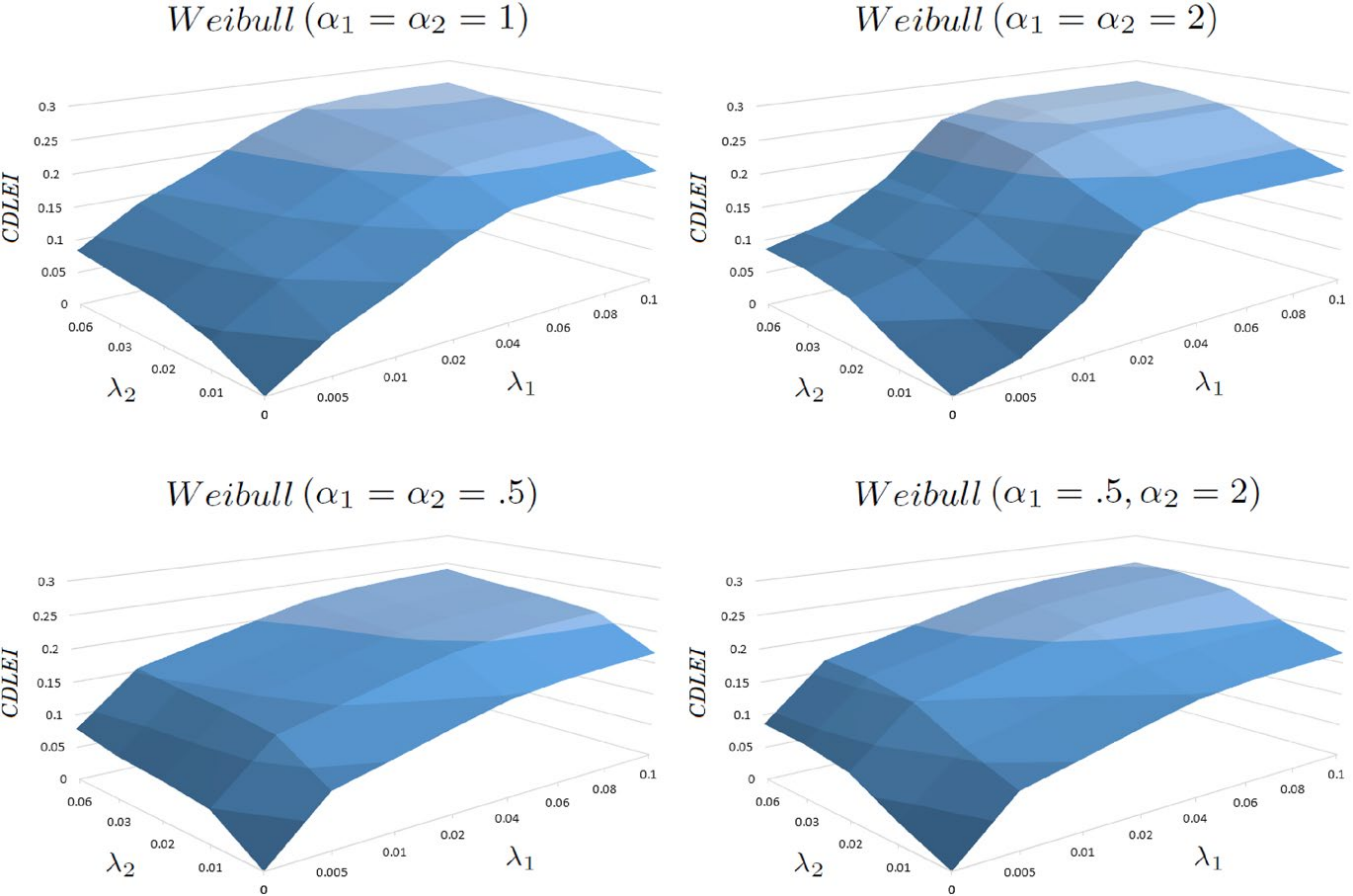
Surface Plots of the CDLEI vs. $\lambda _1$ and $\lambda _2$

Table 2 illustrates the CDLEI results, from age 0, for some select values of the respective cure (scale) parameters $\lambda _1$ and $\lambda _2$ for values of shape parameters $\alpha _1$ and $\alpha _2$ equal to 0.5, 1, and 2. It is felicitous for comparisons to be done at age 0 rather than at any other age so that the potential benefits of a cure would apply to the largest possible cohort (however, there may be special circumstances when it might make sense to use a different age as the reference point). Many attributes of Table 2 are noteworthy, which can also be appreciated graphically by considering Fig. 1. First, note that the predicted increase in life expectancy from birth is 0.26381 years if both Diabetes and HIV were immediately eliminated as possible causes of death. Second, note that if Diabetes were immediately cured, but HIV realized no cure improvement over the lifetime duration under consideration, then the model predicts an immediate 0.17727 years of life expectancy improvement. Conversely, if HIV realized an immediate cure but there was no Diabetes cure on the horizon for the long term, a CDLEI of about 0.08603 years would be projected. Third, notice how swiftly the maximum CDLEI is reached when $\alpha _1 = \alpha _2 = 2$ compared to the memoryless cure functions where $\alpha _1 = \alpha _2 = 1$. When $\lambda _1$ and $\lambda _2$ reach values a little above 0.06, the life expectancy improvement from age 0 essentially reaches its maximum and will not appreciably increase with larger forces of cure above this value. The opposite is true when $\alpha _1 = \alpha _2 = .5$, where the maximum CDLEI is realized much more slowly. Finally, for the mixed case where $\alpha _1 = .5$ and $\alpha _2 = 2$, the CDLEI statistics seem to more closely resemble the $\alpha _1 = \alpha _2 = .5$ case than the others cases, presumably owing to the fact that mortality rates for Diabetes are so much higher than for HIV (even in the face of significantly higher HIV cure probability).

To get our bearings a little, a value of 0.07 for $\lambda _1$ or $\lambda _2$, when $\alpha = 1$ for example, would correspond to statements such as:

$\bullet$ about an 80% chance of a cure within the next 23 years

$\bullet$ about a 50% chance of a cure within the next 10 years.

These statements may seem entirely plausible to experts in Diabetes and/or HIV research. If that were true, then a *cure threshold* is essentially realized and it would be justifiable to use the traditional cause-deleted approach of simply taking these causes of deaths out of consideration (since the maximum CDLEI is more or less achieved when $\lambda = 0.07$ and $\alpha = 1$ in both cases). If these two statements are deemed overly ambitious, then the CDLEI will very much depend on what values of $\lambda _1$ and $\lambda _2$ analysts are willing to assume (not to mention which $\alpha$ to assume, which for the Weibull cure model will likely be a little in excess of 1).

## Conclusion and future work

In this paper we have proposed models that can incorporate multiple distributions for the probability that cures for specific causes of death can be realized over time, for the purpose of estimating a more accurate cause-deleted life expectancy improvement. Despite the fact that it is difficult to model a cure event that has not yet happened, the proposed methodology is a vast improvement over the prevailing practice of simply assuming the cause in question is removed with certainty. Various factors, including stage of development in research for a cure, interim successes, and even the overall amount of scholarship that is being invested in the cure of a specific cause of death, can all influence the postulated probability of a cure that experts in the field could realistically consider.

There are several avenues of further research that flow from this present work. First, a natural extension of the theory presented in this paper would be to consider cases where there is a “partial cure”. By partial cure, we mean that there are treatments available that prolong life, even though a full cure is not yet realized. A partial cure will translate into a higher life expectancy, effective immediately. As noted by Manton et al. [15], a cause-delay model provides a mechanism for incorporating the likely effects of medical innovation on survival, and this could prove useful for computing a superior CDLEI statistic. A second area to consider is the possibility of dependent cure time random variables. In practice, there can easily be dependence in the probabilities of cures being realized for certain causes of death that are naturally related: for example, the probability of a cure for breast cancer is likely significantly correlated to the probability of a cure for other types of cancers, such as leukemia. The use of copula models to account for dependent cure distributions might very well be the best approach to consider.

## Appendix

### Proof of Theorem 1

Let $(F_{j_1},F_{j_2}, \cdots , F_{j_n})$ be the probability that cures have been found for *n* causes $j_1,j_2,\cdots j_n$. Let *P*(*n*) be such that the *n* dimensional cure model satisfies the equation

7.1 $$\begin{aligned} h_n^*={\bar{h}}_n F^n {\mathbf{1}}_{2^n\times 1}' \end{aligned}$$

where $F^n$ is the Kronecker product of *n* diagonal matrices of order 2 such that each ${{\mathbf{F}}_{\mathbf{k}}}, k=1,2,\cdots ,n$ matrix has the form as in Equation (4.6) so that $F^n$ is a diagonal matrix of the order $2^n$, ${\mathbf{1}}'$ is a $2^n \times 1$ vector of ones. Also, ${\bar{h}}_n = (h^{(i_{2^n})},h^{(i_{2^{n}-1})} , \cdots , h^{(i_2)}, h^{(i_1)} )$ , which has $2^n$ hazard functions with various linear combinations of causes, $-j_1,-j_2,\cdots -j_n$, and $h^{i_1} = h^{}$, the hazard consisting of all risks.

For ${n=1}$, we have $2^1=2$ terms of hazards in the vector ${\bar{h}}_1$ and a single matrix in the Kronecker product of the form $$\begin{bmatrix} F_{j_{1}}&{}0\\ 0&{} 1-F_{j_{1}} \end{bmatrix}$$ such that

7.2 $$\begin{aligned} h_1^*={\bar{h}}_1 F^1 {\mathbf{1}}_{2 \times 1}' = (h^{(-j_1)}, h^{( \tau )})\begin{bmatrix} F_{j_{1}}&{}0\\ 0&{} 1-F_{j_{1}} \end{bmatrix} (1,1)' \end{aligned}$$

which is clearly equal to Equation (3.1). Hence the statement *P*(1) is true.

Now assume that the statement *P*(*n*) is true for $n=m$ as given in Equation (7.1). That is, assume

7.3 $$\begin{aligned} h_m^*={\bar{h}}_m F^m {\mathbf{1}}_{2^m \times 1}' , \end{aligned}$$

where $F^m$ is the Kronecker product of *m* diagonal matrices of order 2, each ${{\mathbf{F}}_{\mathbf{k}}}, k=1,2,...,n$ has the form as in Equation (4.6) so that $F^m$ is a diagonal matrix of order $2^m$.

For ${n=m+1}$, we can formulate the hazard function as

7.4 $$\begin{aligned} h_{m+1}^* = {\tilde{h}}_m F_{i_{m+1}} + h_{m}^* [1- F_{i_{m+1}}] \end{aligned}$$

by following Equations (4.3), where $h_{m}^*$ is same as in the statement where *P*(*m*) is assumed true and ${\tilde{h}}_{m} =(h^{(r_{2^m})},h^{(r_{2^{m}-1})} , \cdots , h^{(r_2)}, h^{(r_1)} )$. Equation (7.4) can be expanded as

7.5 $$\begin{aligned} h_{m+1}^*= & {} (h^{(r_{2^m})},h^{(r_{2^{m}-1})} , \cdots , h^{(r_2)}, h^{(r_1)} ){{{\mathbf{F}}_{{\mathbf{m}}}} \otimes \cdots \otimes F_2 \otimes F_1}{\mathbf{1}}_{2^m \times 1}' F_{i_{m+1}} \nonumber \\&+(h^{(i_{2^m})},h^{(i_{2^{m}-1})} , \cdots , h^{(i_2)}, h^{(i_1)}) {{{\mathbf{F}}_{{\mathbf{m}}} \otimes \cdots \otimes {\mathbf{F}}_{\mathbf{2}} \otimes {\mathbf{F}}_{\mathbf{1}}}}{\mathbf{1}}_{2^m \times 1}'[1- {F_{i_{m+1}}}]. \end{aligned}$$

Next, we wish to show that Equation (7.5) has the even more simplified form

7.6 $$\begin{aligned} h_{m+1}^*={\bar{h}}_{m+1} F^{m+1} {\mathbf{1}}_{2^{m+1}\times 1}' \,, \end{aligned}$$

with $h_{m+1}$ being the vector of $m+1$ hazards, ${\mathbf{1}}'$ the $2^{m+1}\times 1$ vector of ones and $F^{m+1}$ the $m+1$ Kronecker products of the $F_{m+1}$ matrices of the form found in Equation (4.6). Now the $m+1$ Kronecker product can be written in the following form:

$$\begin{aligned} F^{m+1} =\begin{bmatrix} F_{i_{m+1}}&{}0\\ 0&{} 1-F_{i_{m+1}} \end{bmatrix} \otimes \underbrace{{\mathbf{F}}_{m} \otimes \cdots \otimes \mathbf{F}_2 \otimes \mathbf{F}_1}_{B}. \end{aligned}$$

Applying Lemma 1 to the Kronecker product of $m+1$ terms, we obtain

$$\begin{aligned} F^{m+1}= & {} \begin{bmatrix} F_{i_{m+1}}I_m&{}{\mathbf{0}}_{2^m}\\ {\mathbf{0}}_{2^m}&{} 1-F_{i_{m+1}} I_m \end{bmatrix} \begin{bmatrix} {{\mathbf{F}}_{m} \otimes \cdots \otimes {\mathbf{F}}_2 \otimes {\mathbf{F}}_1}&{}{\mathbf{0}}_{2^m}\\ {\mathbf{0}}_{2^m} &{} {{\mathbf{F}}_{m} \otimes \cdots \otimes {\mathbf{F}}_2 \otimes {\mathbf{F}}_1} \end{bmatrix} \\= & {} \begin{bmatrix} {{\mathbf{F}}_{m} \otimes \cdots \otimes {\mathbf{F}}_2 \otimes {\mathbf{F}}_1}&{}{\mathbf{0}}_{2^m}\\ {\mathbf{0}}_{2^m} &{} {{\mathbf{F}}_{m} \otimes \cdots \otimes {\mathbf{F}}_2 \otimes {\mathbf{F}}_1} \end{bmatrix} \begin{bmatrix} F_{i_{m+1}}I_m&{}{\mathbf{0}}_{2^m}\\ {\mathbf{0}}_{2^m}&{} 1-F_{i_{m+1}} I_m \end{bmatrix}, \end{aligned}$$

since the two matrices are commutative. The notation ${\mathbf{0}}_{2^m}$ represents the zero matrix of order $2^m$ so that the each matrix in the product is of order $2^{m+1}$.

Next we perform the vector multiplication of $F^{m+1}{\mathbf{1}}'$ to obtain a part of the general statement as in Equation (7.6):

$$\begin{aligned} F^{m+1}{\mathbf{1}}' = \left[ \begin{array}{c|c} {{\mathbf{F}}_{m} \otimes \cdots \otimes {\mathbf{F}}_2 \otimes {\mathbf{F}}_1}&{}{\mathbf{0}}_{2^m}\\ \hline \\ {\mathbf{0}}_{2^m} &{} {{\mathbf{F}}_{m} \otimes \cdots \otimes {\mathbf{F}}_2 \otimes {\mathbf{F}}_1} \end{array}\right] \begin{bmatrix} F_{i_{m+1}} \\ \vdots \\ F_{i_{m+1}} \\ \hline \\ 1-F_{i_{m+1}} \\ \vdots \\ 1-F_{i_{m+1}} \end{bmatrix}, \end{aligned}$$

where there are $2^m$ of $F_{i_{m+1}}$ and $2^m$ of $1-F_{i_{m+1}}$. By performing the complete multiplication, we get the hazard function

$$\begin{aligned} h_{m+1}^*={\bar{h}}_{m+1} \left[ \begin{array}{c|c} {{\mathbf{F}}_{m} \otimes \cdots \otimes {\mathbf{F}}_2 \otimes {\mathbf{F}}_1}&{}{\mathbf{0}}_{2^m}\\ \hline \\ {\mathbf{0}}_{2^m} &{} {{\mathbf{F}}_{m} \otimes \cdots \otimes {\mathbf{F}}_2 \otimes {\mathbf{F}}_1} \end{array}\right] \begin{bmatrix} F_{i_{m+1}} \\ \vdots \\ F_{i_{m+1}} \\ \hline \\ 1-F_{i_{m+1}} \\ \vdots \\ 1-F_{i_{m+1}} \end{bmatrix}, \end{aligned}$$

where ${\bar{h}}_{m+1} =(h^{(r_{2^n})},h^{(r_{2^{n}-1})} , \cdots , h^{(r_2)}, h^{(r_1)} |h^{(i_{2^n})},h^{(i_{2^{n}-1})} , \cdots , h^{(i_2)}, h^{(i_1)})$, $h^{(i_1)} = h^{}$ the complete risk, $h^{(r_{2^n})},h^{(r_{2^{n}-1})} , \cdots , h^{(r_2)}, h^{(r_1)}$ are newly incorporated $2^m$ hazards due to the additional variable which gives various combinations of mixture of hazard functions without component variables and corresponding marginals. Now this vector can be written in the form

$$\begin{aligned} {\bar{h}}_{m+1}= & {} (h^{(r_{2^n})},h^{(r_{2^{n}-1})} , \cdots , h^{(r_2)}, h^{(r_1)} |0,\cdots , 0) \\&+ ( 0,\cdots ,0 |h^{(i_{2^n})},h^{(i_{2^{n}-1})} , \cdots , h^{(i_2)}, h^{(i_1)}) \\= & {} ({\overline{h}}_{r} |{{\overline{\mathbf{0}}}}) + ({{\overline{\mathbf{0}}}} |{\overline{h}}_i) \end{aligned}$$

where $${{\overline{\mathbf{0}}}}$$ represents a vector of zeros, $${\overline{h}}_{r}$$ is a representation for the collection of $$h^{(r_{2^n})},h^{(r_{2^{n}-1})} , \cdots , h^{(r_2)}, h^{(r_1)},$$ and $${\overline{h}}_i$$ is the notation used for $$h^{(i_{2^n})},h^{(i_{2^{n}-1})} , \cdots ,$$$$h^{(i_2)}, h^{(i_1)}$$. Then, the new hazard function can be expressed as

$$\begin{aligned} h_{m+1}^*= & {} ({\overline{h}}_{r} |{{\overline{\mathbf{0}}}}) F^{m+1}\mathbf{1 }' + ({{\overline{\mathbf{0}}}} |{\overline{h}}_i) F^{m+1}\mathbf{1 }'\\= & {} ({\overline{h}}_{r} |{{\overline{\mathbf{0}}}})\left[ \begin{array}{c|c} {{\mathbf{F}}_{m} \otimes \cdots \otimes {\mathbf{F}}_2 \otimes {\mathbf{F}}_1}&{}{\mathbf{0}}_{2^m}\\ \hline \\ {\mathbf{0}}_{2^m} &{} {{\mathbf{F}}_{m} \otimes \cdots \otimes {\mathbf{F}}_2 \otimes {\mathbf{F}}_1} \end{array}\right] \begin{bmatrix} F_{i_{m+1}} \\ \vdots \\ F_{i_{m+1}} \\ \hline \\ 1-F_{i_{m+1}} \\ \vdots \\ 1-F_{i_{m+1}} \end{bmatrix} \\ \\&+({{\overline{\mathbf{0}}}} |{\overline{h}}_i) \left[ \begin{array}{c|c} {{\mathbf{F}}_{m} \otimes \cdots \otimes {\mathbf{F}}_2 \otimes {\mathbf{F}}_1}&{}{\mathbf{0}}_{2^m}\\ \hline \\ {\mathbf{0}}_{2^m} &{} {{\mathbf{F}}_{m} \otimes \cdots \otimes {\mathbf{F}}_2 \otimes {\mathbf{F}}_1}\end{array}\right] \begin{bmatrix} F_{i_{m+1}} \\ \vdots \\ F_{i_{m+1}} \\ \hline \\ 1-F_{i_{m+1}} \\ \vdots \\ 1-F_{i_{m+1}} \end{bmatrix}. \end{aligned}$$

Equation (7.5) can be obtained by performing the multiplication for the partitioned matrices, and we get $2^{m+1}$ terms in the expansion of the general form. Hence it is true for $n=m+1$ and so $P(m+1)$ is true. By the principle of mathematical induction, *P*(*n*) is true for all $n\in {\mathbb {N}}/\{0\}$, the set of all natural numbers. $\square$

## Footnotes

1 In this paper, we will use the terms decrement, cause, competing risk and risk interchangeably and treat them as being mutually equivalent.
